# Transcription Factors and Markers Related to Epithelial–Mesenchymal Transition and Their Role in Resistance to Therapies in Head and Neck Cancers

**DOI:** 10.3390/cancers16071354

**Published:** 2024-03-29

**Authors:** Marta Pawlicka, Ewelina Gumbarewicz, Ewa Błaszczak, Andrzej Stepulak

**Affiliations:** Department of Biochemistry and Molecular Biology, Medical University of Lublin, 1 Chodzki Street, 20-093 Lublin, Poland; martapawlicka991@gmail.com (M.P.); ewelina.gumbarewicz@umlub.pl (E.G.); ewa.blaszczak1@umlub.pl (E.B.)

**Keywords:** head and neck cancers, HNC, head and neck squamous cell carcinoma, HNSCC, epithelial–mesenchymal transition, EMT, transcription factors, markers, chemoresistance, radioresistance

## Abstract

**Simple Summary:**

Head and neck squamous cell carcinoma is one of the most common cancers that arises in the upper aerodigestive tract. Patients suffering from this cancer have a high mortality risk, mainly due to local recurrence, resistance to chemo- and radiotherapy, and metastasis. The more aggressive behavior of this tumor is associated with epithelial–mesenchymal transition, a process described in both physiological, primarily during embryonic development, and pathological situations, including the progression of other types of tumors. Epithelial-to-mesenchymal transition is governed by various transcription factors that regulate target gene expression and play a role in the resistance to contemporary head and neck cancer therapies. This review presents the current knowledge of the main transcription factors involved in mesenchymal conversion and discusses their role in head and neck squamous cell carcinoma treatment. The main protein markers associated with this cancer type are also presented.

**Abstract:**

Head and neck cancers (HNCs) are heterogeneous and aggressive tumors of the upper aerodigestive tract. Although various histological types exist, the most common is squamous cell carcinoma (HNSCC). The incidence of HNSCC is increasing, making it an important public health concern. Tumor resistance to contemporary treatments, namely, chemo- and radiotherapy, and the recurrence of the primary tumor after its surgical removal cause huge problems for patients. Despite recent improvements in these treatments, the 5-year survival rate is still relatively low. HNSCCs may develop local lymph node metastases and, in the most advanced cases, also distant metastases. A key process associated with tumor progression and metastasis is epithelial–mesenchymal transition (EMT), when poorly motile epithelial tumor cells acquire motile mesenchymal characteristics. These transition cells can invade different adjacent tissues and finally form metastases. EMT is governed by various transcription factors, including the best-characterized TWIST1 and TWIST2, SNAIL, SLUG, ZEB1, and ZEB2. Here, we highlight the current knowledge of the process of EMT in HNSCC and present the main protein markers associated with it. This review focuses on the transcription factors related to EMT and emphasizes their role in the resistance of HNSCC to current chemo- and radiotherapies. Understanding the role of EMT and the precise molecular mechanisms involved in this process may help with the development of novel anti-cancer therapies for this type of tumor.

## 1. Introduction

Each year, over 800,000 patients are diagnosed with head and neck cancer (HNC) [1], which has a mortality rate close to 50% [2]. HNC is a heterogeneous group of tumors located in the upper aerodigestive tract, i.e., the oral cavity, oropharynx, larynx, hypopharynx, nasopharynx, nasal cavity and paranasal sinuses, and salivary glands. Many histological types of HNC havd been distinguished. Here, we focus on the most common histological type of head and neck cancer, namely, squamous cell carcinoma (HNSCC), which accounts for over 90% of HNCs [3,4].

A higher risk of developing HNSCC is associated typically with tobacco use, alcohol consumption, Epstein–Barr virus (EBV) or human papillomavirus (HPV) infection, in particular infection with HPV types 16 and 18, and other factors [5]. Currently, the morbidity of HPV-related HNSCC is increasing, while the incidence rate of HNSCC related to heavy use of tobacco and alcohol is slowly decreasing [6]. Therapy planning requires multidisciplinary insight and individual adjustment. In the early stages of HNSCC (stages I-II out of IV), surgery or radiotherapy (RTH) is usually used as a single treatment method, depending on the tumor localization, surgical accessibility, and experience of a particular healthcare center. However, combined treatment, that is chemotherapy (CHT), RTH, and surgery, is needed for more than half of patients, especially those with the more advanced tumor stages. According to the American Joint Committee on Cancer (AJCC), in oropharyngeal cancer staging, HPV status is included, and stage I disease is considered to involve nodes that are no larger than 6 cm and ipsilateral [7]. Strikingly, HPV-positive patients respond better to RTH and CHT than HPV-negative patients [6]. Nasopharyngeal cancer staging also differs from other HNSCCs, as these cancers are usually not resected; therefore, the staging does not include pathologic classification, and RTH is a primary treatment for these tumors [7,8].

Platinum-based chemotherapy, paclitaxel (Taxol), docetaxel, and 5-fluorouracil (5-FU) are administered to patients with stages III/IV HNSCC as systemic treatments [6,8]. Cetuximab, a monoclonal antibody that targets the epidermal growth factor receptor (EGFR), applied together with cisplatin (cis-diamminedichloroplatinum(II); CDDP) and 5-FU, is used in recurrent/metastatic HNSCC treatment [3]. Recently, the PD-1 receptor protein-targeting drugs nivolumab and pembrolizumab have also been used in patients with recurrent/metastatic HNSCCs treated previously with platinum-containing CHT. CDDP resistance develops in about one-third of cases, whereas resistance to 5-FU is present in about 40% of HNSCCs [9].

Chemo- and radiotherapy resistance, associated with poor patient prognosis, remain unsolved problems for many cancer types, including HNSCC. In comparison to other cancers, HNSCC is a more local disease with primary tumor and neck lymph node metastases and relatively less distant metastasis formation. However, if the formation of distant metastasis occurs, it is also associated with a very poor prognosis [10,11,12]. Several pathways have been suggested to be involved in acquired and intrinsic resistance development, dissemination, and recurrence of HNSCC and have been recently reviewed in [2,13,14]. Here, we highlight the current knowledge of the process of epithelial–mesenchymal transition (EMT) in HNSCC. The main EMT protein markers in HNSCC are summarized. EMT is a key regulatory process associated with cancer metastasis, which is governed by transcription factors [15]. We therefore discuss the main transcription factors related to EMT and emphasize their role in resistance to chemo- and radiotherapy in patients with HNSCC. Understanding the role of EMT and the molecular mechanisms involved in this process may help with the development of novel anti-cancer therapies for HNSCC.

## 2. A Brief Overview of Epithelial–Mesenchymal Transition

**Figure 1:**
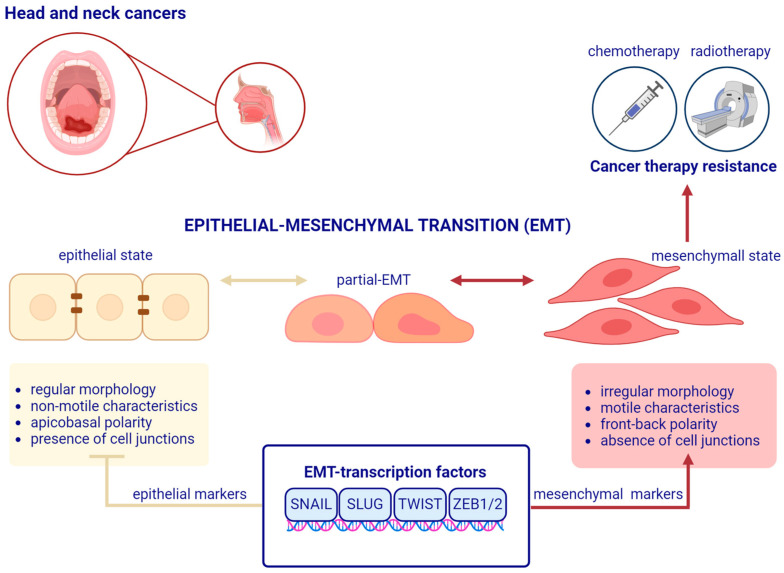
A schematic representation of the epithelial–mesenchymal transition process in head and neck cancer. During the epithelial–mesenchymal transition (EMT) process, non-motile, cuboidal epithelial cells lose their characteristics, namely, apicobasal polarity and the connections between them. Before reaching the mesenchymal state, they exhibit a hybrid epithelial/mesenchymal phenotype (partial-EMT process). The mesenchymal state, in which cells are motile with an irregular morphology and front-back polarity, is associated with therapy resistance in head and neck cancer. EMT may be reversible, a process termed mesenchymal–epithelial transition (MET). EMT is regulated by multiple factors, including EMT transcription factors, such as SNAIL, SLUG, TWIST, and ZEB1/2, that influence the expression of epithelial or mesenchymal markers. Created with BioRender.com.

Epithelial–mesenchymal transition (EMT) (Figure 1), first described by Elizabeth Hay in the late 1970s in the chick embryo model [16,17], is a highly conserved, complex, multistep process involved in physiological and pathological events such as embryogenesis (type 1), wound healing, tissue fibrosis (type 2), and cancer progression (type 3) [18,19,20]. EMT is induced by signals from the local microenvironment, including growth factors and cytokines, hypoxia, and extracellular components. Tumor epithelial cells that undergo EMT acquire mesenchymal properties by losing cell junction proteins, by losing polarity from apicobasal to front-back, by obtaining stem cell properties, and through changes in cytoskeletal composition [21,22]. In cancer cells, during the EMT process, the expression of mesenchymal markers (e.g., vimentin, N-cadherin, and vitronectin) and proteins, such as matrix metalloproteinases, is activated. In turn, the levels of epithelial markers (e.g., E-cadherin, cytokeratin, claudin, and occludin) essential for cell-cell adhesion, epithelial properties, and tight junction maintenance decrease. As a consequence of EMT, the invasive capacity and motility of cancer cells increase [15,23,24].

Expression of epithelial and mesenchymal markers is driven by key transcription factors, such as TWIST1, TWIST2, SLUG, SNAIL, ZEB1, and ZEB2, and it is regulated at molecular and cellular levels via different signaling cascades, including receptor-mediated intracellular signaling pathways [15]. The EMT process is also controlled by post-translational modifications (PTMs) and epigenetic factors, including phosphorylation, DNA modifications, histone methylation/acetylation, and altered expression of non-coding RNAs (ncRNAs) such as microRNAs (miRNAs), circular RNAs, (circRNA), and long non-coding RNAs (lncRNAs) [25,26,27,28,29].

Recently, the EMT International Association (TEMTIA) highlighted that the EMT process comprises a partially transformed hybrid epithelial and mesenchymal phenotype, the so-called partial EMT (p-EMT) [30,31]. p-EMT results in the co-expression of epithelial and mesenchymal markers [32,33] (described below) and in stem cell-like properties such as a self-renewal ability and strengthened differentiation potential of cancer cells [24]. p-EMT subpopulations of cells existing in invasive tumors have been linked to metastatic potential and a poor prognosis in patients with HNSCC [34,35,36]. The reverse process, known as mesenchymal–epithelial transition (MET) is characterized by the acquisition of epithelial properties in cells with EMT-induced phenotypes. During this process, mesenchymal-like cells reorganize their cytoskeleton and obtain apical-basal polarity; cell-cell adhesion increases, therby resulting in an organized epithelium. This process occurs during embryonic development, for instance, kidney morphogenesis, cardiac growth, and somite formation. MET is also associated with cancer, particularly metastasis, where mesenchymal cells revert to epithelial ones to regain proliferating abilities at secondary sites [30].

## 3. The Main EMT Markers in Head and Neck Squamous Cell Carcinoma

**Figure 2:**
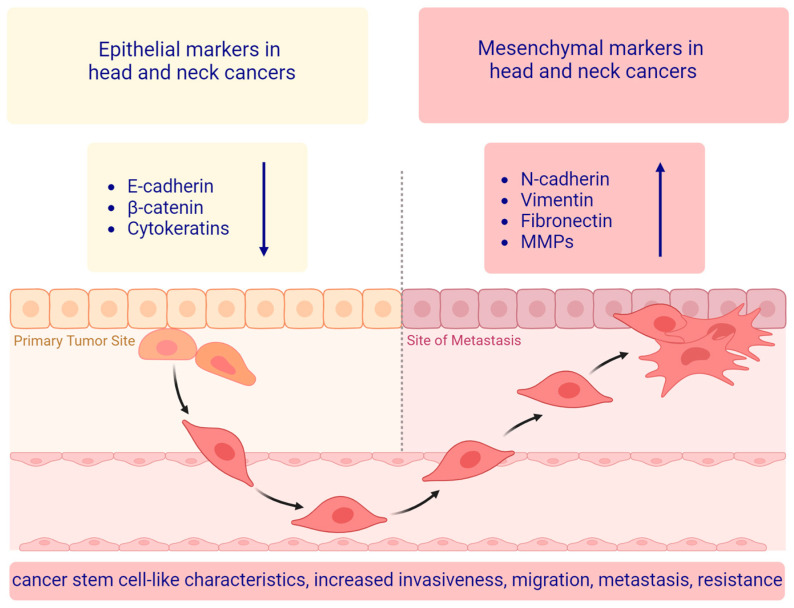
The main protein markers and key changes in head and neck cancers associated with epithelial–mesenchymal transition (EMT). The expression levels of epithelial markers such as E-cadherin, β-catenin, and cytokeratins are decreased, while the expression levels of mesenchymal markers, such as N-cadherin, vimentin, fibronectin, along with other proteins associated with the mesenchymal state such as MMPs (matrix metalloproteinases), are increased. Cancer cells undergoing the EMT process acquire cancer stem cell-like characteristics associated with a mesenchymal state to facilitate their migration, invasion, and metastasis. This is associated with therapy resistance in this type of cancer. ↑—indicate an increase in the expression level; ↓—indicate a decrease in the expression level. Created with BioRender.com.

Several protein markers have been described to identify and characterize cells undergoing EMT [37]. These proteins are crucial for the changes that occur during this process, and their expression is correlated with the transition from an epithelial to a mesenchymal phenotype (Figure 2). They are classified as epithelial markers, e.g., E-cadherin and β-catenin, and mesenchymal markers, e.g., vimentin, fibronectin, and other EMT-associated proteins, e.g., matrix-metalloproteinases. Molecular and cellular changes associated with the EMT process include a progressive shift from epithelial polarity to loss of apical-basal polarity and enhancement of front-back polarity, changes in marker expression, such as cadherin variants from E-cadherin to N-cadherin, and adoption of a mesenchymal morphology as a result of suppression of cell-cell contacts [38,39,40].

### 3.1. Epithelial Markers

There are several proteins associated with epithelial cell phenotypes that are often used to describe the epithelial characteristics of cancer cells in HNSCC. The most common epithelial markers with a decrease in HNSCC include E-cadherin, β-catenin, and cytokeratins. Other proteins, such as claudin 4 and 7 and components of desmosomes, are also associated with the epithelial identity and show a decrease in HNSCC [41,42].

#### 3.1.1. E-Cadherin

E-cadherin is a member of the cadherin protein family, specifically found on the surface of epithelial cells, which plays a crucial role in cell-cell adhesion. Mandal and colleagues [43] documented increased levels of activated Src protein tyrosine kinase in HNSCC cells, together with the reduced expression levels of E-cadherin and the increase in the mesenchymal marker vimentin. This phenotype correlates with an aggressive tumor phenotype, characterized by invasiveness, and the presence of lymph node metastasis [43]. Accordingly, Nijkamp and colleagues [44] demonstrated a potential correlation between the loss of E-cadherin and the presence of vimentin, indicating increased migratory capabilities in tumor cells and an increased risk of metastasis in patients with HNSCC. These studies also highlight an essential role of the cadherin-β-catenin complex in adherens junctions, emphasizing that its downregulation leads to the disruption of cell-cell contacts, tissue instability, and the onset of cancer invasion. The phenomenon known as the “cadherin switch” (marked by the decrease in E-cadherin and increase in N-cadherin mesenchymal marker expression) propels tumor cells to acquire mobility and invasiveness, resulting in the loss of their epithelial integrity [45,46].

#### 3.1.2. β-Catenin

As aforementioned, β-catenin is a subunit of the cadherin protein complex that is primarily considered an epithelial marker. β-catenin anchored to the cell membrane plays a role in facilitating cell-cell adhesion by participating in adherens junction formation via binding to E-cadherin and α-catenin. Despite its involvement in cell adhesion as a key component of adherens junctions, it also functions as an intracellular signal transducer in the Wnt signaling pathway. The stability of cytoplasmic β-catenin is determined by its phosphorylation status. In the absence of Wnt signaling, β-catenin located in the cytoplasm is targeted to the proteasome for degradation through the ubiquitin-mediated proteolytic pathway upon interaction with adenomatous polyposis coli (APC) protein and axins and phosphorylation by the axin-bound casein kinase 1 (CK1) and glycogen synthase kinase-3 beta (GSK-3β) degradation complex [47]. In the presence of Wnt signaling ligands, low-density lipoprotein receptor-related proteins 5 and 6 (LRP5 and LRP6) undergo phosphorylation, leading to the recruitment of dishevelled cytoplasmic proteins to the plasma membrane and subsequent inactivation of the degradation complex. In turn, cytoplasmic β-catenin is stabilized and translocated to the nucleus. When β-catenin reaches and accumulates in the nucleus, it binds to DNA-binding transcription factors, such as T-cell and lymphoid enhancer factors (TCF-LEF) to regulate EMT-related gene expression. β-catenin is abnormally expressed in precancerous lesions and many cancers [48], including HNSCC [49,50,51], and the changes in its expression indicate that β-catenin can be considered a marker of EMT. Recently, Reed and colleagues [52] showed that β-catenin/CREB-binding protein (CBP) activation of the mechanistic target of rapamycin complex 1 (mTORC1; previously referred to as the mammalian target of rapamycin) signaling promotes partial epithelial–mesenchymal states.

#### 3.1.3. Cytokeratins

Other proteins found in epithelial cells are cytokeratins, intermediate filament proteins that provide cell-to-cell adhesion support and are also common markers for epithelial differentiation. Cytokeratins form heterodimers between two classes, namely, acidic type I and basic type II cytokeratins. Fifty-four cytokeratins are encoded by the human genome, with 28 genes encoding type I and 26 genes encoding type II cytokeratins. The expression of various cytokeratins has been linked to different types of cancer. One of the promising cytokeratins for diagnosis and therapy of HNSCC is cytokeratin 8 (CK8). High levels of CK8 have been found in tumor cells with a malignant phenotype, including HNSCC [53,54,55,56]. Antibodies against CK8 have also been detected in the serum of patients diagnosed with HNSCC [57,58]. Cytokeratin 19 (CK19) has also been proposed to be a biomarker for the presence of highly invasive HNSCCs prone to metastasis. Strong immunoreactivity for CK19 is observed in metastatic lymph nodes of HNSCC patients with optimal cut-off points of the labeling index (LI) between 5% ≤ LI < 77% and LI ≥ 77%. Disease-specific survival curves indicate worse prognostic outcomes for those with LI ≥ 77% [59]. Dysregulation of epithelial differentiation has also been noticed in the context of cytokeratin 4 (CK4) and cytokeratin 13 (CK13) expression in HNSCC. Downregulation of CK4 and CK13, the primary pair of differentiation-associated cytokeratins in oral keratinocytes, suggests an alteration in epithelial differentiation in HNSCC and oral epithelial dysplasia, which is a premalignant condition characterized by abnormal changes in the oral epithelial tissue. These keratins could prove valuable for pathological diagnosis [60]. In another study of 67 oral mucosa specimens, CK13 and CK17 were analyzed in different stages of oral malignancies. Normal epithelia showed 100% CK13 positivity and 0% CK17 positivity, while carcinoma in situ (CIS) and HNSCC displayed 100% CK17 positivity and reduced CK13 levels. CK17 and CK13 positivity was observed only in distinct keratinization areas of HNSCC and CIS, indicating a reciprocal relationship between CK17 and CK13 expression levels, with CK17 emergence linked to malignancy [61]. The combined loss of expression of CK13 and another protein marker involucrin is associated with a poor prognosis of HNSCC (tongue cancer) [62].

### 3.2. Mesenchymal Markers

#### 3.2.1. Vimentin

One of the markers of the EMT mechanism that shows increased expression in HNSCC is vimentin [63,64]. Vimentin is a type III intermediate filament protein [65,66] that occurs not only in eukaryotic mesenchymal cells but also in cancer cells [67]. Its main physiological function is to maintain the cellular structure by forming intermediate filaments, resulting in the formation of an intracellular network that performs several important functions, such as the maintenance of cell shape, mechanical resistance, organelle transport, and the maintenance of nuclear integrity. Furthermore, vimentin participates in many other processes that involve the formation of complexes with several cell signaling molecules [68,69]. Interestingly, even though vimentin is a cytoskeletal protein, it has been shown to be involved in the apoptotic process. During the progression of apoptosis, vimentin is cleaved by caspase-3, caspase-7, and caspase-6, resulting in cytoskeletal collapse. This is considered the basis of the morphological changes occurring in cells undergoing apoptosis. Direct phosphorylation of vimentin by AKT1 serine/threonine protein kinase (a known cancer-promoting kinase) protects it from caspase-mediated cleavage and blocks apoptotic progression [70,71]. Vimentin interacts with phosphorylated ERKs (extracellular signal-regulated kinases), which belong to the mitogen-activated protein (MAP) kinase (MAPK) family and protect it from dephosphorylation [67].

In the context of cancer cells, changes in the expression or structure of vimentin may affect their function. In these cases, excessive expression of vimentin is fundamental in migration, invasion, and motility, promoting resistance to stress conditions [72]. Overexpression of vimentin contributes to several cancers’ progression, invasion, and/or metastasis, including breast [73,74], ovarian [75], prostate [76,77], lung [78], colorectal cancers [79], tumors of the central nervous system [80] and gastrointestinal tract [81], malignant melanoma [82,83], and also head and neck squamous cell carcinoma [44,63]. Moreover, vimentin upregulation is related to poor prognosis and clinical outcome, e.g., in acute myeloid leukemia [70].

Nijkamp and colleagues [44] assessed vimentin expression in HNSCC together with epithelial marker E-cadherin in patient biopsies using immunohistochemistry (IHC). Vimentin expression was present in 46% of the biopsies and was observed in tumor cells surrounding blood vessels. High vimentin fractions corresponded to tumors, where the EMT process has occurred. The decreased E-cadherin and increased vimentin expression were proposed to be linked to the increased mobility of tumor cells, leading to a higher risk of metastasis in patients diagnosed with HNSCC [44]. Liu and colleagues [63] identified high vimentin expression in the HN12 cell line derived from HNSCC using transcriptomic microarray, followed by Western blotting, reverse transcription PCR (RT-PCR), and immunofluorescence (IF). This was correlated with lymph node metastasis in vitro. Knockdown of vimentin hindered the migratory and invasive capabilities of highly metastatic HN12 cells. Importantly, in patients with HNSCC, vimentin expression was significantly higher in those having lymph node metastases than in those without lymph node metastasis, as shown by IHC staining [63].

Vimentin participates in metastatic progression via cytoskeletal reorganization, and it is classified as a mesenchymal marker due to its capacity to upregulate β1-integrin and downregulate the expression of the junction protein E-cadherin [84]. The molecular mechanism of vimentin regulation depends on miRNA-138 (miR-138) and miRNA-141 (miR-141), which participate in cell migration, adhesion, and signaling processes. Overexpression of miR-141 inhibits EMT progression in transforming growth factor (TGF)-β1-treated HK-2 cells by the maintenance of high E-cadherin expression levels and low vimentin expression levels [85,86]. Downregulation of miR-138 is also observed in HNSCC and other cancer types. Previous studies suggest that the downregulation of miR-138 is associated with mesenchymal-like cell morphology, enhanced cell migration, and invasion. Furthermore, studies have demonstrated that these miR-138-induced changes are accompanied by a marked reduction in E-cadherin expression and enhanced vimentin expression [87].

#### 3.2.2. Fibronectin-1

Other studies have shown that EMT correlates with resistance to epidermal growth factor receptor (EGFR) inhibitors in various solid tumors, including HNSCC. Haddad and colleagues [88] used real-time quantitative PCR and immunoblotting to characterize the expression of E-cadherin, vimentin, fibronectin, and delta-crystallin enhancer-binding factor 1 (deltaEF1) across a panel of HNSCC-derived human cell lines to decipher mechanisms underlying the resistance to erlotinib. This small tyrosine kinase inhibitor has been developed with a specific focus on inhibiting the activity of EGFR in cancer [89]. In cells sensitive to erlotinib treatment, E-cadherin levels are high, while vimentin, fibronectin-1, and deltaEF1 levels are low. Conversely, in erlotinib-resistant cells, fibronectin-1 and deltaEF1 levels are high, while E-cadherin levels are low [88]. The fibronectin-1 encoding gene (*FN1*), whose expression is increased in cancer cells, shows EMT plasticity and is often used as a biomarker of the mesenchymal phenotype [90]. This gene is an essential component of the extracellular matrix (ECM) [91]. Elevated levels of cellular fibronectin are found in HNSCC patients [92]. Small interfering RNA-mediated silencing of the *FN1* gene has demonstrated the involvement of *FN1* in the viability, adhesion, migration, and invasive properties related to other tumors as well, e.g., thyroid cancer cells [93].

Transforming growth factor-beta 1 (TGF-β1), a cytokine and signaling molecule that plays a crucial role in various cellular processes, including cell growth and differentiation, activates EMT in HNSCC via the so-called SMA and MAD-related protein-dependent pathway (the SMAD-dependent pathway) [94,95,96]. Moreover, it plays an essential role in the formation of tumor buds that separate from primary tumors in HNSCC through activation of the ZEB1 transcription factor [97]. TGF-β1 also acts in cooperation with the nuclear transcription factor PRRX1 to regulate the EMT, tumor cell migration, and invasion in HNSCC [98]. PRRX1 overexpression leads to cadherin alterations with decreased levels of E-cadherin, increased levels of N-cadherin, and increased levels of vimentin. Additionally, it leads to the upregulation of other transcription factors such as SLUG and ZEB2. SLUG plays a central function in the induction of the EMT and has the role of a prognostic marker associated with poor clinical outcomes in HNSCC. In HNSCC, SLUG is responsible for the switch from E-cadherin to N-cadherin under hypoxic conditions and after hypoxia-inducible factor 1-alpha (HIF-1α) overexpression in cancer cell lines [99].

#### 3.2.3. N-Cadherin

In primary HNSCC, the SLUG and HIF-1α transcription factor expression levels correlate with an increase in N-cadherin levels in patients with poor overall survival [99,100]. The SLUG-dependent increase in N-cadherin levels and decrease in E-cadherin levels in oral cancer cells are consistent with adherens junction reorganization and loss of desmosomes [101]. A recent study demonstrates that the so-called “cadherin-switch”, characterized by the loss of E-cadherin and the emergence of de novo expression of N-cadherin in the invasion front of recurrent HNSCC, is a consistent histological feature. This feature remains unchanged from the initial presentation of the tumor to its recurrence, irrespective of the type of adjuvant therapy administered during the primary tumor treatment. The absence of E-cadherin expression in recurrent HNSCC independently poses a higher risk for poor survival outcomes and holds prognostic value [102].

### 3.3. Other Protein Markers Associated with the EMT

Other biomolecules linked to cell adhesion or manifesting increased expression in HNSCC, include matrix metalloproteinases, α-catulin, or vinculin. They influence HNSCC tumorigenesis possibly by increasing cell mobility, cell cycle progression, and EMT, being therefore potential biomarkers or promising targets for the treatment of HNSCC [103,104].

#### Matrix Metalloproteinases

Matrix metalloproteinases (MMPs) are a group of calcium-dependent zinc-containing proteolytic enzymes [105]. Metalloproteinases-encoding genes are expressed across most cells, including those of the stationary type (such as macrophages, fibroblasts, keratinocytes, Langerhans dendritic cells, myocytes, endothelial cells, microglial cells, and neurons) and cells occurring in the inflammatory infiltrate (such as leukocytes, monocytes, and T lymphocytes) [106]. MMPs play significant roles in cellular regeneration, apoptosis, angiogenesis, and many other essential functions, including both normal development and pathological processes [107]. In recent years, matrix MMPs have been well established to play a key role in the complex mechanism of cancer progression [108]. Their main function is to digest components of the extracellular matrix and vascular basement membrane, facilitating tumor growth, cell migration, and tumor invasion. They also enhance the formation of metastases and angiogenesis within the tumor. Studies have shown a clear tendency toward increased expression levels of the tested metalloproteinases and their inhibitors in laryngeal cancer compared to healthy tissues [109,110].

Examples of protein markers with increased expression in HNSCC are matrix metalloproteinase-9 (MMP-9) and matrix metalloproteinase-2 (MMP-2), termed gelatinases. These are enzymes responsible for the degradation of collagen IV, the main building block of the basement membrane structure. By degrading the basement membrane and extracellular matrix components, they are essential factors in the process of tumor invasion [111]. Canel and colleagues [112] proposed that focal adhesion kinase (FAK) increases the invasion potential of HNSCC by facilitating cell motility and the production of MMP-2. This, in turn, was suggested as a potential strategy for therapeutic interventions. Aparna and colleagues [113] investigated the expression of MMP-2 and MMP-9 in HNSCC (tongue cancer; stages I and II) and correlated their expression with local recurrence, distant dissemination of tumor cells, and survival outcomes. Their results showed a positive correlation between the gelatinases and metastasis of this tumor similar to previous reports with MMP-9 being more specific and better indicating patient prognosis [113]. Importantly, the aggressiveness of HNSCC is partly related to the overexpression of histone deacetylase 6 (HDAC6) and bromodomain-containing protein 4 (BRD4). They promote cell proliferation, enhance the survival of tumor cells, and reduce apoptosis by activating the AKT protein kinases and nuclear factor kappa-light-chain-enhancer of activated B cells (NF-κB) signaling pathways. Moreover, they enhance tumor necrosis factor α-induced (TNF-α-induced) migration and invasion by elevating the expression of MMP-2, MMP-9, and MT-MMP-1 [114].

## 4. EMT-Related Transcription Factors and Treatment Resistance in Head and Neck Squamous Cell Carcinoma

**Figure 3:**
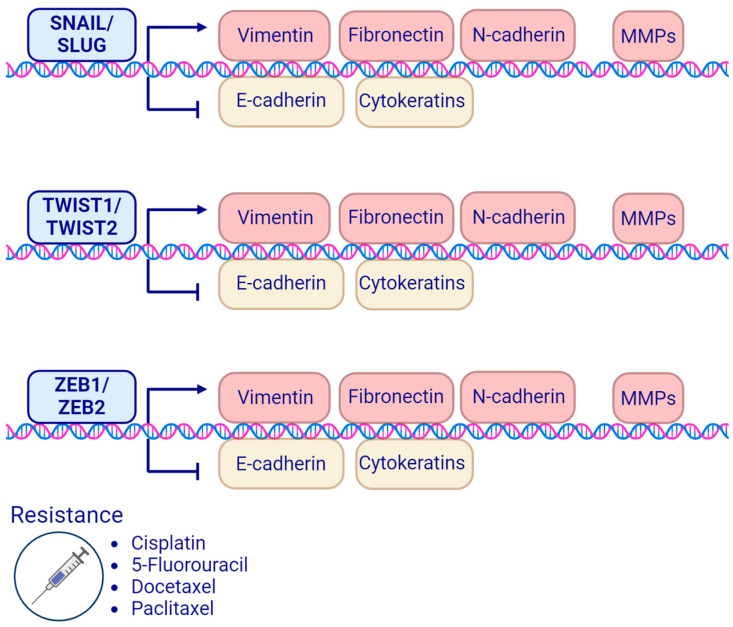
The main epithelial–mesenchymal transition-related transcription factors (EMT-transcription factors) in head and neck cancers and their influence on the epithelial and mesenchymal markers with examples of therapy resistance. Created with BioRender.com.

Regulation of the EMT process is complex, and it is initiated and orchestrated by transcription factors (TFs). The major EMT-TFs in HNSCC (Figure 3) include the SNAIL family proteins, SNAIL (*SNAI1* gene) and SLUG (*SNAI2* gene); the TWIST family proteins, TWIST1 (*TWIST1* gene) and TWIST2 (*TWIST2* gene); and the zinc finger E-box binding (ZEB) homeobox family proteins ZEB1 (*ZEB1* gene) and ZEB2 (the *ZEB2* gene is also known as *SIP1*) [15]. EMT-TFs repress epithelial markers, e.g., E-cadherin expression, support the development of metastases and tumor dissemination, and attenuate the response to therapy, which correlates with poor prognosis [115,116].

### 4.1. SNAIL Family Transcription Factors

The SNAIL family transcription factors involved in the EMT include SNAIL, SLUG, and a SNAIL-related transcription factor SMUC encoded by the *SNAI3* gene. These are evolutionarily conserved proteins that in vertebrates take part in mesoderm formation. They are characterized by the presence of zinc finger regions [117]. SNAIL family proteins are involved in the EMT process via the upregulation of mesenchymal markers like fibronectin-1 and the downregulation of epithelial markers [118]. Although the role of SNAIL and SLUG in the EMT process and carcinogenesis in HNSCC has been well described, the role of SMUC in HNSCC remains unknown. Mice lacking SMUC exhibit nearly typical characteristics, suggesting that SMUC is not essential for mouse embryonic development [119].

#### 4.1.1. SNAIL Transcription Factor

Overexpression of SNAIL in human HNSCC and oral epithelial cells leads to the EMT process, which is confirmed by elevated mesenchymal markers, a decreased capacity to form tight spheroids, increased dissemination capacity, and resistance to erlotinib, a tyrosine kinase inhibitor (TKI) targeting the epidermal growth factor receptor (EGFR) [120]. EGFR is overexpressed in up to 90% of HNSCC and plays a role in the pathogenesis of HNSCC as well as various other cancers, in which it is a potential molecular target. Other TKIs include gefitinib, which is approved for non-small cell lung carcinoma (NSCLC) but has also been proposed as a therapeutic agent in HNSCC. However, the clinical benefit of gefitinib therapy in HNSCC patients is still inconsistent. In vitro studies indicate that the resistance to gefitinib treatment in HNSCC may be mediated by the downregulation of EGFR, leading to the activation of the AKT/GSK-3β/SNAIL pathway and promoting EMT [121].

Beyond the influence of gene expression, the mechanical properties of cancer cells play a crucial role in cancer dissemination. This is influenced, among other factors, by cellular forces that enable cancer cells to navigate through narrow pores in the ECM and create microchannels, thereby enhancing directional migration. In a mouse model, the biomolecular and biochemical properties of lymph node metastatic and non-metastatic HNSCC cells were examined. Lymph node metastatic HNSCC cells demonstrated increased invasion capabilities compared to non-metastatic ones. These observed properties were linked to a more elongated morphology of cancer cells, displaying a more mesenchymal phenotype, and were characterized by increased longitudinal strain of the cell nuclei, leading to stronger cell traction force and softer nuclear stiffness. These biomechanical changes were found to strongly correlate with elevated SNAIL expression [122]. In vitro studies on SNAIL-transfected HNSCC cells (SAS and HSC-4) have shown lower E-cadherin protein levels and an increase in their migration and invasion abilities in comparison to control cells. Additionally, the expression level of cancer stem cell (CSC) surface markers in SNAIL-transfected cell lines (HNSCC-SNAIL), namely, cell surface glycoprotein CD44 and aldehyde dehydrogenase 1 (ALDH1), were elevated. SNAIL- transfected cells showed enhanced chemoresistance for CDDP [123]. In another study, HNSCC cells (CNE2 nasopharyngeal carcinoma cells) exhibited the acquisition of a CSC-like phenotype through the EMT process once transfected with *SNAI1*. The increased expression of stem cell-associated TFs, including NANOG, OCT4, and BMI1, along with CSC markers CD44 and CD133, demonstrated a correlation with enhanced invasion, migration abilities, and radioresistance both in vitro and in vivo. Taken together, these findings confirm SNAIL’s ability to induce the EMT process and CSC phenotype in HNSCC cells [124], potentially contributing to increased resistance to CHT and RTH. Zhao and colleagues [125] aimed to evaluate the expression and possible roles of SNAIL in HNSCC using comprehensive bioinformatics approaches, followed by histological methods. The overexpression of SNAIL was assessed in a cohort of a tissue microarray (47 cases) and a cohort of HNSCC patients (68 cases). The authors concluded that the increased expression of SNAIL could potentially facilitate lymph node metastasis via intricate molecular processes and serve as a prognostic marker in HNSCC [125].

The nucleotide excision repair (NER) process is a crucial cellular defense mechanism against, for instance, the cytotoxic effects of platinum-based chemotherapeutic agents. An enzyme playing a pivotal role in this process is excision repair cross-complementation group 1 (ERCC1). A positive correlation has been observed between ERCC1 mRNA levels and resistance to platinum-based chemotherapy. In HNSCC cell lines, the induction of SNAIL overexpression has been linked to increased ERCC1 expression, while the knockdown of SNAIL results in downregulated ERCC1 expression, correlating with higher sensitivity to CDDP. Furthermore, the same study demonstrated elevated expression levels of ERCC1 and SNAIL in tumor samples from HNSCC patients (*n* = 72) who received cisplatin-based chemotherapy compared to normal epithelium samples from the same patients. The study revealed a correlation between ERCC1 and SNAIL expression and elevated CDDP resistance [126].

#### 4.1.2. SLUG Transcription Factor

The expression of SLUG is higher in HNSCC tumor cells compared to normal oropharyngeal mucosa and correlates with elevated EMT marker expression. Interestingly, recent studies indicate that SLUG expression levels strongly correlate with partial EMT, whereas other EMT-TFs are associated rather with complete EMT [127,128,129]. The prevalence of lymph node metastases in HNSCC is one of the most important prognostic factors. In vitro studies have shown that the upregulation of SLUG expression favors the invasion and metastasis to lymph nodes of HNSCC cells via the Wnt/β-catenin signaling pathway [130]. SLUG may be a potential inducer and regulator of partial EMT in HNSCC, as demonstrated in clinical cohorts and cell line studies. In the same study, it was observed that lower SLUG expression in HNSCC patients correlated with improved disease-free survival. Additionally, SLUG was associated with enhanced invasion and resistance to irradiation [129]. In another study, it was found that the likelihood of primary radiotherapy or chemotherapy (RTH/CHT) failure in patients with SLUG-positive tumors was 3.6 times higher compared to SLUG-negative patients with HNSCC. The survival of SLUG-positive HNSCC patients was higher in the group treated with upfront surgery/concurrent systemic therapy (PORT) compared to primary RTH/CHT. In conclusion, SLUG expression levels have been suggested as a potential predictive biomarker useful in treatment planning for HNSCC patients [131]. Moreover, elevated CDDP chemoresistance may correlate with the overexpression of SLUG, enhancing stemness phenotypes in HNSCC. CSCs represent a population of cancer cells characterized by self-renewal and multipotent differentiation. Cancer cells acquire CSC properties through the EMT process [132].

In vitro studies have demonstrated that SLUG knockdown leads to a reduction in stem-cell properties, such as the ability for sphere formation, downregulation of CSC markers CD44 and aldehyde dehydrogenase (ALDH), and an increase in the expression of stemness genes (*OCT4*, *SOX2*, *NANOG*). The downregulation of SLUG expression is associated with increased sensitivity of HNSCC to CDDP by disrupting stem-cell properties. Conversely, SLUG overexpression is linked to enhanced chemoresistance in cancer cells through the upregulation of ABC transporter genes, tumor initiation, and invasion in a xenograft model [132].

The SLUG-SOX9 transcription factor axis is involved in the regulation of CSC properties. SLUG protects SOX9 stability [133] from proteasomal degradation, a process in which proteins are tagged with a small protein ubiquitin and are targeted for degradation by a large protein complex—proteasome [134,135]. Inhibition of both SLUG and SOX9 suppresses, e.g., lung cancer metastasis in a mouse model [133]. The SLUG modulator includes TGF-β1, whose stimulation in HNSCC leads to the overexpression of SLUG in vitro and is associated with resistance to CDDP [136]. Interestingly, reciprocal expression between SLUG and SNAIL has been reported. SLUG small interfering RNA (siRNA) significantly increases SNAIL expression in HNSCC-derived cells, while SNAIL siRNA also increases the level of SLUG expression. In addition, TGF-β1 upregulates the expression of both SNAIL and SLUG. Hence, SLUG or SNAIL siRNA alone only partially alleviates malignant phenotypes in the presence of TGF-β1, but the combined action of SLUG and SNAIL siRNAs suppresses them remarkably. Thus, targeting both SLUG and SNAIL simultaneously, rather than individually, holds promise as a new therapeutic approach for HNSCC [137].

### 4.2. TWIST Family Transcription Factors

The TWIST family transcription factors include the TWIST1 and TWIST2 proteins belonging to the larger basic–helix–loop–helix (bHLH) family. The *TWIST1* and *TWIST2* genes show a high level of similarity in their genetic sequences, and the proteins are highly conserved [138]. The *TWIST1* gene, primarily identified in *Drosophila melanogaster*, has been shown to be essential for embryogenesis. *Drosophila* embryos lacking this gene fail to undergo normal gastrulation, produce no mesoderm, and die displaying a morphology with a “twisted” appearance [139,140]. Twist2 was initially identified in a yeast two-hybrid (Y2H) screen, and the protein was originally termed Dermo1 for how it was expressed in the developing dermis of the mouse embryos [141]. Throughout mammalian embryonic development, TWIST2 is present in mesodermal tissues, but it is expressed temporarily and later than TWIST1 [138]. The members of the TWIST TF family also play a key role in dermal fibroblast regulation, but the precise functional mechanism is still unknown [142]. Both transcription factors are shown to be involved in cancer progression and metastasis [143,144,145,146]. These may be promoted via the stimulation of the EMT process [15].

TWIST1 expression is significantly associated with a reduced level of E-cadherin in HNSCC. Its overexpression, which correlates with a poor prognosis in HNSCC patients, may constitute a prognostic factor for this group [147,148]. This could be linked to TWIST1-mediated resistance to CHT, including the multidrug resistance (MDR) mechanism. One of the proteins involved in MDR is multidrug resistance protein 1 (MDR1), a P-glycoprotein (P-gp) responsible for drug exclusion, which may be regulated by TWIST1. In stable hypopharyngeal cancer cell lines characterized by the overexpression of TWIST1, elevated MDR1/P-gp activity is observed. Conversely, silencing TWIST1 expression results in a decrease in MDR1/P-gp expression. In the same study, the chemosensitivity of cancer cells to paclitaxel (Taxol) increased with TWIST1 and MDR1/P-gp upregulation and decreased with their downregulation. Interestingly, TWIST1 overexpression attenuated apoptosis by promoting the apoptosis repressor BCL-2 protein and downregulating apoptosis promoters BAX, caspase-3, and caspase-9. One of the mechanisms of action of Taxol in cancer cells is the induction of Ca^2+^ release. Elevated activity of TWIST1 modulates Taxol-triggered apoptosis by decreasing intracellular free cytosolic Ca^2+^ [149]. In vitro studies in liver cancer (HepG2 cells) also yield similar results: TWIST1 upregulates MDR1 activity, supporting efflux pump efficiency and consequently reducing the concentration of chemotherapeutics such as CDDP, 5-FU, and doxorubicin in cancer cells. Conversely, the knockdown of TWIST1 sensitizes liver cancer cells to these chemotherapeutics [150].

The anticancer action of chemotherapeutics, ionizing radiation, and ligand engagement of death receptors, such as those of the tumor necrosis factor receptor (TNF-R) family, is based on the induction of programmed cell death (PCD). NF-kB TFs play an essential role in controlling apoptosis. The activation of NF-kB interferes with apoptosis, potentially allowing cancer cells to survive, leading to tumor proliferation and chemoresistance. It has been suggested that TWIST1 and TWIST2 play crucial roles in NF-kB-dependent chemoresistance. It was found that TWIST1 protects NF-kB activity, blocks daunorubicin-induced PCD, interferes with the p53 and p19ARF pathways, and suppresses BCL-2 inhibitory phosphorylation [151].

### 4.3. ZEB Homeobox Family Transcription Factors

Members of the zinc finger E-box-binding (ZEB) homeobox family, namely, ZEB1 and ZEB2, originally discovered in *Drosophila melanogaster*, play a physiological role in controlling processes such as renal and limb development during embryogenesis. However, they are also implicated in the EMT process in various types of cancers [152,153]. ZEB1 and ZEB2 have been demonstrated to play an important role in promoting cancer cell migration, invasion, and metastasis across various tumor types, including bladder, breast, and pancreatic cancers [12,154]. The involvement of ZEB1 and ZEB2 in HNSCC and their potential contribution to therapeutic resistance remains an ongoing area of investigation.

#### 4.3.1. ZEB1 Transcription Factor

ZEB1, encoded by the *ZEB1* gene (also known as *δEF1*, *AREB6*, *ZFHEP*, *ZFHX1A*, *BZP*, *NIL-2-A*, and *DeltaEF1*), is a 1117 amino acid protein. It takes part in the differentiation of bone tissue, smooth muscle tissue, and neural tissue. Except for embryogenesis, ZEB1 plays a role in multidrug resistance, proliferation, metastasis, and cancer development [155].

In head and neck cancer studies in vitro, erlotinib-resistant HNSCC lines have been shown to express high levels of ZEB1 and low levels of E-cadherin. Furthermore, increased invasiveness and migratory capabilities have been observed. In turn, the knock-down of ZEB1 in erlotinib-resistant cell lines results in increased sensitivity to erlotinib [88]. The chimeric IgG1 monoclonal antibody (mAb) cetuximab, which specifically binds to the EGFR, is recommended when added to radiotherapy or platinum-based chemotherapy for patients with locally advanced HNSCC. However, the response to cetuximab as a single agent is low (6–13%). Resistance to cetuximab may be associated with the upregulation of EMT. A significant increase in the expression of EMT-related genes, including ZEB1 and TWIST1, has been observed in patients with HNSCC, after cetuximab administration. The authors demonstrated that in addition to its anti-tumor properties, cetuximab prompts substantial alterations within the tumor. These modifications include changes in both genes and proteins associated with the extracellular matrix and cancer-associated fibroblasts (CAFs) [156]. Furthermore, it was demonstrated that the overexpression of ZEB1 and B lymphoma Mo-MLV insertion region 1 (BMI-1; a protein that belongs to a Polycomb group family of proteins) in patients with head and neck cancers was also associated with resistance to chemotherapy. In cisplatin-resistant HNSCC cell lines, there was an increase in the expression level of ZEB1, while histone deacetylase 1 (HDAC1) was downregulated. This observed mechanism may be associated with the CSC population, which was increased in conjunction with chemoresistance [157]. Targeting ZEB1 by mi-RNA-101 in oral squamous cell carcinoma resulted in the inhibition of proliferation, apoptosis resistance, migration, and invasion in vitro. It also suppressed tumor growth and lung metastasis in a xenograft model (metastasis mouse model). Moreover, it was shown that ZEB1 expression is higher in these tissues in comparison to non-cancerous ones. This negatively correlated with the miR-101 level in oral squamous cell carcinoma [158]. An example of the unclear role of EMT in HNSCC was shown in a study where a livestock antibiotic salinomycin induced apoptosis, increased chemosensitivity, activated AKT signaling, and surprisingly induced the expression of EMT markers, including vimentin, and the transcription factors ZEB1 and SNAIL, while decreasing E-cadherin expression [159].

#### 4.3.2. ZEB2 Transcription Factor

ZEB2, encoded by the *ZEB2* gene (also known as *KIAA0569*, *SIP1*, *ZFHX1B*, and *ZFX1B*), is a 1214 amino acid protein [160]. Its elevated expression has been found in HNSCC, and its inhibition is associated with lower viability, migration, and invasion in vitro. It has been demonstrated that lower expression of the EMT markers, specifically vimentin and N-cadherin, correlates with the downregulation of ZEB2. Silencing ZEB2 also results in a decrease in the activity of the MMP-2/MMP-9 enzymes that degrade collagen IV and, consequently, facilitate the migration and invasive properties of HNSCC. While this mechanism may potentially play a role in the development of chemoresistance, further studies are needed for a comprehensive understanding. It has been shown that ZEB2 expression in HNSCC tissues is upregulated compared to normal tissues. Additionally, the association between ZEB2 and clinicopathological parameters has been analyzed in a cohort of 76 patients with operable tumors. ZEB2 and β-catenin expression were related to a later tumor stage, inducing a less differentiated tumor phenotype and reducing overall survival [161]. In another study, poorer overall survival in HNSCC patients was associated with co-expression of TWIST1 and ZEB2 [162].

The amount of available data regarding the role of ZEB2 in chemoresistance in HNSCC is limited. However, studies on other cancers indicate that ZEB2 may be involved in resistance to therapy. These include, for instance, research related to gastric cancer, where downregulation of ZEB2 expression in cisplatin-resistant gastric cancer cell lines resulted in elevated sensitivity to cisplatin [163,164]. In ovarian cancer cells, upregulation of ZEB2 determines cisplatin resistance [165]. Overexpression of circular RNA circ_0007534 downregulates miRNA-625 (miR-625) and upregulates ZEB2 expression, which is associated with paclitaxel resistance in endometrial cancer [166]. In nasopharyngeal carcinoma cells (NPC), targeting ZEB2 by miRNA-203 (miR-203) reduces resistance to CDDP by modulating tumor stemness signals both in vitro and in vivo. Expression of stemness factors including BMI1, SOX2, NANOG, and OCT4 correlates with ZEB2 expression. Moreover, knocking down *ZEB2* leads to the suppression of migration and tumor invasion in NPC-derived cell lines, increased E-cadherin expression, and lower N-cadherin expression [167].

Moreover, the knockdown of ZEB1/ZEB2 expression in HNC-CD133-positive cells causes suppression of CSC-like properties, which in turn is related to increased drug resistance and loss of self-renewal ability. On the other hand, overexpression of ZEB1/ZEB2 in HNC-CD133-negative cells leads to the sphere-forming ability and increases the percentage of cell surface glycoprotein CD44-positive cells and side population cells. By using siRNA of ZEB1 and ZEB2 in xenograft tumors, it was shown that the tumor growth was reduced as was the rate of distant metastasis formation, suggesting that inhibition of ZEB1/ZEB2 expression was effective [168]. There is a definite need for further research on the role of ZEB2 in the development of resistance to therapy in HNSCC.

## 5. Summary of the Current Knowledge of EMT, Transcription Factors, and Markers in HNSCC

EMT is a process in which epithelial cells lose their characteristics and acquire properties of mesenchymal cells. The partial and the reversal nature of the EMT process introduces diversity within cancer cell clusters, resulting in cells displaying different degrees of epithelial and mesenchymal characteristics and promoting heterogeneity [169]. Thus, hybrid epithelial–mesenchymal cells, which promote the formation of metastasis have been found in circulating tumor cells from patients with different types of cancers. Epithelial–mesenchymal crosstalk, that is, the interaction between the supportive tissue surrounding the tumor (tumor stroma), including associated fibroblasts and epithelial cancer cells, has also been found in HNSCC. Epithelial–mesenchymal crosstalk induces a hybrid phenotype in HNSCC, characterized by the simultaneous expression of vimentin and cytokeratin, that is associated with chemoresistance and increased cell proliferation and migration. This phenotype is also observed in the tissue biopsies from head and neck cancer patients [170]. Taken together, EMT is a key step in cancer progression.

A substantial number of TFs related to EMT have been identified. The main TFs that orchestrate the EMT process in HNSCC include the SNAIL, SLUG, TWIST1, TWIST2, ZEB1, and ZEB2 proteins. Importantly, the expression of these TFs promotes the migration, invasion, proliferation, and survival of HNSCC cells (for a systematic review see [171]). EMT-TFs regulate the expression of both epithelial (e.g., E-cadherin, β-catenin, and cytokeratins) and mesenchymal markers (e.g., vimentin, fibronectin 1, and N-cadherin) in HNSCC. In recent years, RNA sequencing (RNA-seq), especially single-cell sequencing, followed by bioinformatics analysis, has been used for transcription profiling and a better understanding of tumor biology. Due to intra-tumoral heterogeneity, RNA-seq has been employed to identify the role of p-EMT in HNSCC. It has been suggested that the p-EMT process may promote local invasion and metastasis. A transcription factor whose expression correlated with p-EMT across HNSCC is SNAIL2 [34]. Using RNA-seq, other studies have determined the subclusters of cancer-associated fibroblasts (CAFs) in HNSCC [172]. Although the available data regarding the examination of the role of EMT-TFs in HNSCC using RNA-seq are still limited, this technique with a combination of bioinformatics approaches seems promising to find EMT regulatory pathways in HNSCC [173,174]. Therefore, inhibiting EMT-TFs could potentially reverse the epithelial–mesenchymal transition in HNSCC. Moreover, the expression levels of other proteins linked to cell adhesion or proteins manifesting increased expression in HNSCC, e.g., matrix metalloproteinases (MMP-2 and/or MMP-9), may serve as potential biomarkers for HNSCC.

Given the heterogeneous nature of this cancer and the diverse mechanisms underlying therapy resistance, it is important to expand the knowledge of the precise molecular mechanisms underlying the EMT process. Targeting EMT is important not only from the perspective of hindering cancer progression, invasion, and metastasis but also from the perspective of improving the response of HNSCC and other cancers to current therapies.

## 6. Conclusions

The diverse and aggressive nature of HNSCC has impeded the discovery of molecular targets for the advancement of targeted therapies. Moreover, the resistance to current therapies, namely chemo- and radiotherapy, along with their associated toxicities represent a huge problem for patients and contribute to poor survival rates. Understanding the molecular mechanisms underlying chemo- and radiotherapy resistance should have a substantial influence on patient outcomes. The more aggressive behavior of HNSCCs is associated with epithelial–mesenchymal transition. The complex process of EMT encompasses numerous transcription factors that influence the expression of target genes and signaling pathways. Despite numerous studies indicating a correlation between therapy resistance in HNSCC and the EMT process, further studies are needed, and many questions remain open. For instance, how could therapies (alone or in combination with radiotherapy, chemotherapy, some other targeted drugs, and/or immunotherapy) be optimized to achieve better patient outcomes while minimizing side effects? How could these treatments be personalized and patient care significantly improved? What are the precise molecular mechanisms governing the EMT process in HNSCC, and how can this knowledge be used to improve diagnosis and therapy for these types of cancer?

## Abbreviations

AJCC	American Joint Committee on Cancer
ALDH	Aldehyde dehydrogenase
APC	Adenomatous polyposis coli
BRD4	Bromodomain-containing protein 4
CAFs	Cancer-associated fibroblasts
CBP	CREB-binding protein
CDDP	Cis-diamminedichloroplatinum(II) (Cisplatin)
CHK1	Checkpoint kinase 1
CHT	Chemotherapy
CK	Cytokeratin
cRNA	Circular RNA
CSC	Cancer stem cells
DDR	DNA damage response
deltaEF1	Delta-crystallin enhancer-binding factor 1
EBV	Epstein–Barr virus
ECM	Extracellular matrix
EGFR	Epidermal growth factor receptor
EMT	Epithelial–mesenchymal transition
ERCC1	Excision repair cross-complementation group 1
FAK	Focal adhesion kinase
5-FU	5-Fluorouracil
HADAC	Histone deacetylase
HIF-1α	Hypoxia-inducible factor 1-alpha
HNC	Head and neck cancer
HNSCC	Head and neck squamous cell carcinoma
HPV	Human papillomavirus
IF	Immunofluorescence
IHC	Immunohistochemistry
LI	Labeling index
lncRNA	Long non-coding RNA
MAP	Mitogen-activated protein
MAPK	Mitogen-activated protein kinase
MDR	Multidrug resistance
MDR1	Multidrug resistance protein 1
MET	Mesenchymal–epithelial transition
miRNA	MicroRNA
MMP	Matrix metalloproteinase
mTORC1	Mechanistic target of rapamycin complex 1
ncRNA	Non-coding RNA
NER	Nucleotide excision repair
NF-κB	Nuclear factor kappa-light-chain-enhancer of activated B cells
NPC	Nasopharyngeal carcinoma cells
NSCLC	Non-small cell lung carcinoma
PCD	Programmed cell death
PTMs	Post-translational modifications
p-EMT	Partial epithelial–mesenchymal transition
P-gp	P-glycoprotein
RMHNC	Recurrent/metastatic head and neck carcinoma
ROCK2	Rho-associated coiled-coil-containing protein kinase 2
RTH	Radiotherapy
RT-PCR	Reverse transcription PCR
siRNA	Small interfering RNA
TCF-LEF	T-cell and lymphoid enhancer factors
TF	Transcription factor
TGF-β1	Transforming growth factor-beta 1
TJ	Tight junction
TKI	Tyrosine kinase inhibitor
TNF-R	Tumor necrosis factor receptor
TNF-α	Tumor necrosis factor α-induced
USP7	Ubiquitin-specific protease 7
ZFH	Zinc finger E-box-binding homeobox protein family
